# The Mammalian Phenotype Ontology as a unifying standard for experimental and high-throughput phenotyping data

**DOI:** 10.1007/s00335-012-9421-3

**Published:** 2012-09-09

**Authors:** Cynthia L. Smith, Janan T. Eppig

**Affiliations:** The Jackson Laboratory, Bar Harbor, ME 04609 USA

## Abstract

The Mammalian Phenotype Ontology (MP) is a structured vocabulary for describing mammalian phenotypes and serves as a critical tool for efficient annotation and comprehensive retrieval of phenotype data. Importantly, the ontology contains broad and specific terms, facilitating annotation of data from initial observations or screens and detailed data from subsequent experimental research. Using the ontology structure, data are retrieved inclusively, i.e., data annotated to chosen terms and to terms subordinate in the hierarchy. Thus, searching for “abnormal craniofacial morphology” also returns annotations to “megacephaly” and “microcephaly,” more specific terms in the hierarchy path. The development and refinement of the MP is ongoing, with new terms and modifications to its organization undergoing continuous assessment as users and expert reviewers propose expansions and revisions. A wealth of phenotype data on mouse mutations and variants annotated to the MP already exists in the Mouse Genome Informatics database. These data, along with data curated to the MP by many mouse mutagenesis programs and mouse repositories, provide a platform for comparative analyses and correlative discoveries. The MP provides a standard underpinning to mouse phenotype descriptions for existing and future experimental and large-scale phenotyping projects. In this review we describe the MP as it presently exists, its application to phenotype annotations, the relationship of the MP to other ontologies, and the integration of the MP within large-scale phenotyping projects. Finally we discuss future application of the MP in providing standard descriptors of the phenotype pipeline test results from the International Mouse Phenotype Consortium projects.

## Introduction

Systematic collection and curation of phenotypic descriptions began in the 1940s as textual synopses for mouse mutants (Snell 1941). Then, maintaining and updating text manually was easy (very few mutants known) and there were no electronic records requiring search mechanisms or computer-parsable formats. Now there are over 24,300 mutant alleles that have been identified in mice^1^ with established phenotypes, representing mutant alleles in over 9,600 genes (Table 1). Also, there are nearly 4,700 QTL (quantitative trait loci) that represent genomic regions associated with particular phenotypic traits. Phenotypic characterization data continue to expand rapidly. The Mouse Genome Informatics (MGI http://www.informatics.jax.org) (Blake et al. 2011; Eppig et al. 2012) database, the primary international database for mouse, adds several hundred new mutant alleles with reported phenotypes to the database each month.

**Table 1:** Mutant allele and phenotype data in the Mouse Genome Informatics database, MGI^a,b,c^

Data type	Count
Total mutant alleles (in ES cell lines and mice)	738,364
Mutant alleles in mice	24,339
Genes with mutant alleles	14,743
Genes with mutant alleles in mice	9,636
Mammalian phenotype ontology (MP) terms	8,744
Genes with phenotype annotations	8,903
Genotypes with phenotype annotations	43,335
Total MP annotations to genotypes	227,169
Human diseases with one or more genotypic mouse models	1,148
Mouse genotypes modeling human diseases	3,668
Quantitative trait loci (QTL)	4,696
Total recombinase (Cre)-expressing transgenes and alleles	1,739

^a^Data as of May 5, 2012, www.informatics.jax.org. New data are added to the MGI database daily; thus, actual counts will be higher than those shown here

^b^Mutant allele counts include spontaneous, induced (e.g., by ENU), and genetically engineered alleles. Transgenes, which are not part of the normal mouse genome, are not included

^c^Mutants present only in ES cell lines versus those created in mice or made into mice from ES cells are distinguished in several table counts. All phenotype-related data refer to mutations present in mice

Researchers increasingly develop sophisticated new mouse models of human disease and analyze phenotypes in mice carrying complex engineered and mutant allele combinations on multiple genetic backgrounds. The unit of annotation for a phenotype, therefore, must be the animal(s) or “whole” genotype assessed, annotated to MP terms and accompanied by key conditional variables (e.g., treatment, age of onset). Thus, the actual number of phenotype-bearing populations far exceeds the number of mutant alleles. Such data maintained by continuous resynthesis of information as descriptive text are (1) impractical to maintain; (2) unreliable to search without structured format and controlled vocabularies, producing false-negative and false-positive search errors; and (3) not amenable to computational analyses.

Large-scale projects to produce a complete set of mutations “for every gene” in the mouse are underway using phenotype-driven mutagenesis approaches [cf. ENU (*N*-ethyl-*N*-nitrosourea)] (Acevedo-Arozena et al. 2008; Clark et al. 2004; Cook et al. 2006; Goldowitz et al. 2004) and gene-driven approaches (cf. gene-trap and gene-knockout programs) (Araki et al. 2009; Austin et al. 2004; Auwerx et al. 2004; Nord et al. 2006). These new data sets and the need to restructure phenotype data representation in MGI prompted transformation of text-based phenotypic descriptions into structured annotations based on the MP, which was initiated concurrently as a phenotype annotation tool in 2001.

Restructuring of MGI’s phenotype data included (a) development of a data model for phenotypes in the MGI relational database; (b) development of the MP ontology (Smith and Eppig 2009; Smith et al. 2004) as the cornerstone for phenotype annotation; (c) application of the MP ontology to ongoing curation of phenotypes in MGI and the retirement of text-based descriptions; (d) development of new, robust access to phenotypes via redesigned web interfaces, tracks on the MGI Mouse Genome Browser (http://gbrowse.informatics.jax.org/cgi-bin/gbrowse/mouse_current), and contribution of data to other genome browser resources such as University of California Santa Cruz (UCSC, http://genome.ucsc.edu), Ensembl (http://www.ensembl.org) and NCBI (National Center for Biotechnology Information, http://www.ncbi.nlm.nih.gov); and (e) development of a human disease view of mouse phenotypes utilizing MGI’s annotations to OMIM (Online Mendelian Inheritance in Man, http://www.omim.org) disease terms.

The significance of the mouse as a model organism, the availability of its fully sequenced genome, and the accessibility of mouse tissues for experimentation at all life-stages invite new applications and exquisite experimental manipulation to address key scientific questions. Integration of experimental data using standard data descriptions and nomenclatures is of paramount importance in maximizing the value of the mouse model system. MGI has loaded large-scale data from ENU phenotyping centers and laboratories and is poised to load new phenotyping data from the developing International Mouse Phenotyping Consortium (IMPC, www.mousephenotype.org), allowing integration of these data with data derived from individual laboratories and the biomedical literature. A common interface to phenotypes in MGI exists that allows critical phenotype and genotype comparisons. Further, alignment of mouse mutant phenotypes with human disease symptoms will aid in identifying mouse genetic models with phenotypic matches as well as the currently captured experimentally demonstrated mouse models for human disease.

## The Mammalian Phenotype Ontology (MP)

**Fig. 1:**
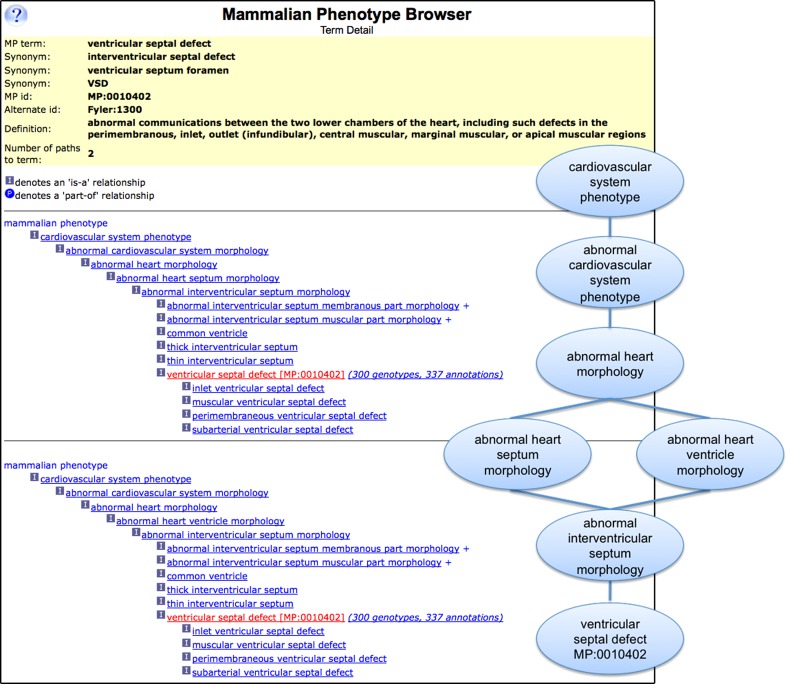
The Mammalian Phenotype Ontology (MP) browser at MGI, showing details for the term “ventricular septal defect” (MP:0010402). *Left* MGI’s MP browser display shows the term name, common synonyms and acronyms, the primary MP ID, alternate IDs, and the term definition. The two term paths are shown as a *hierarchial tree*, with paths listed in multiple sequential graphs. Each term in the MP browser is followed by a link to MGI genotypes associated with that term or any children of that term. *Right* A graphical representation of the DAG structure for the term “ventricular septal defect.” The two alternate paths from the node “cardiovascular system phenotype” are shown

The MP is the workhorse for standardizing phenotypic descriptions in mouse, rat, and other mammals. The MP is a “precomposed” ontology, structured as a DAG (directed acyclic graph) and using phenotype terms recognized by research biologists and clinicians that include simple compound concepts (e.g., liver hyperplasia, MP:0005141) and aggregate concepts (e.g., glomerular crescent, MP:0011506) (Fig. 1).

The MP is a flexible, expandable tool that can grow to accommodate the anticipated rapid increase in phenotyping data, can be applied to maximize precision and breadth of user phenotype searches, and can facilitate an efficient curation stream of incoming phenotype data. By annotating phenotypes from these data sets using MP, the standardization and concurrent retrieval of terms is achieved. This stands in contrast to natural language text, where there is no restriction on the variation of term names, descriptors, or grammar, confounding data integration and limiting effectiveness of data searches.

As of May 2012, the MP contains 8,744 terms describing morphological, physiological, and behavior anomalies. The top nodes are organized into 27 categories representing biological systems, mortality terms, and behavior, with abnormal morphological and physiological system terms at the next node level. Phenotype data can be annotated at any point along the structure, depending on the detail available from information sources. Each term is distinct and defined, aiding both curators and users in selecting the appropriate term for their needs. In addition, attributes and relationships among the terms are described in the form of a DAG (Fig. 1). This allows more flexibility than that of a simple tree, since each term can have multiple relationships to broader parent terms and more specific child terms. The more specific terms are subsumed by parent terms as one moves up the graph, which allows for more complete grouping, searching, and analysis of annotated data.

Multiple resources provide browser formats for viewing the MP, including the Ontology Lookup Service (OLS, http://www.ebi.ac.uk/ontology-lookup/ontologyList.do), Bioportal (http://bioportal.bioontology.org/ontologies), and MGI’s MP browser (http://www.informatics.jax.org/searches/MP_form.shtml). Figure 1 shows a sample page from MGI’s MP Browser for the phenotype term ventricular septal defect (MP:0010402). Each term in the MP has a unique term name, unique accession ID, synonyms, and a definition. In MGI’s MP Browser, the relationship between parent and child terms is visualized by indentation of each successive level of the hierarchy. Where a term has multiple parents, each path from the upper-level term to the term of interest displays as a separate hierarchy, thus effectively flattening the DAG structure for web viewing. The MP file in OBO format is available for download from the MGI ftp site (ftp://ftp.informatics.jax.org/pub/reports/index.html#pheno); it is also available in OBO and OWL formats from the Open Biomedical Ontologies (OBO, http://www.obofoundry.org) foundry site, OLS, and Bioportal.

The MP is a dynamic ontology, actively used and developed by those annotating phenotypes in mouse and other species. Requests for new terms, term revisions, and suggestions for structural organization modifications to the MP are frequently proposed by curators and user groups. Suggestions for improvement and additions from the community are submitted through the Open Biomedical Ontologies Mammalian Phenotype Requests tracker system at SourceForge (https://sourceforge.net/tracker/?atid=1109502&group_id=76834) or by email to pheno@jax.org.

Expansion of the MP ontology and review of its hierarchical structure occurs in collaboration with new phenotype annotation projects when the need for additional granularity of terms is anticipated. In addition, collaborative review of particular systems by expert editors together with subject area specialists helps create terms and structures that are intuitive and useful to those communities. Recent additions and revisions include the respiratory system, renal/urinary system, and cardiovascular system (with significant structural reorganization) that expanded the MP by 714 terms. To accommodate data being generated by large-scale phenotyping efforts at the Wellcome Trust Sanger Institute (hereafter, Sanger Institute) Mouse Genetics Program (http://www.sanger.ac.uk/mouseportal) and from the EUMORPHIA (Brown et al. 2005; Mandillo et al. 2008) and EUMODIC (Beck et al. 2009; Morgan et al. 2010) European large-scale phenotyping efforts, MP added 38 new population-level lethality terms. These lethality terms also will support data forthcoming from the IMPC projects. Furthermore, 196 new MP homeostasis terms now describe the results of phenotype pipeline tests generated by these centers. When new MP terms are added or revised from these annotation projects or from user requests, relevant existing phenotype annotations at MGI are triggered for review and revised to reflect the new terminology as appropriate.

Along with cardiovascular system term revisions, Fyler codes (Keane et al. 2006), a systematic, hierarchical classification of congenital heart disease (see example in Fig. 1), were included as secondary IDs to the primary MP ID. Fyler codes align the MP to current standards of the cardiac disease research community and its representation in the research and clinical literature. These codes are consistent with the International Pediatric and Congenital Cardiac Codes (IPCCC, http://www.ipccc.net) and enable users to search for congenital heart defects using these codes, IDs, or term names, with comprehensive retrieval of information.

## Applying the MP to phenotype annotations

A number of resources use the MP to describe abnormal phenotypes (see Table 2), including MGI, the Rat Genome Database (RGD, http://rgd.mcw.edu), Online Mendelian Inheritance in Animals (OMIA, http://omia.angis.org.au/home), the Sanger Institute Mouse Genetics Program, MRC Harwell’s MouseBook (http://www.mousebook.org), Europhenome (http://www.europhenome.org), and the IMPC, among others. In addition, the MP is used by mouse repositories to annotate phenotype data (or reflect downloaded MGI phenotype data) for describing available mouse strains and stocks. These include the Jackson Laboratory Repository (JAX^®^ Mice, http://jaxmice.jax.org), the European Mouse Mutant Archive (EMMA, http://www.emmanet.org), and the Mutant Mouse Regional Resource Centers (MMRRC, http://www.mmrrc.org), among others.

**Table 2:** URLs referenced in this article (Those preceded by an asterik (*) incorporate MP terms for phenotype data)

Resource	URL
*AnnotQTL	http://annotqtl.genouest.org
Bioportal	http://bioportal.bioontology.org/ontologies
Chemical Entities of Biological Interest (ChEBI)	http://www.ebi.ac.uk/chebi
Collaborative Cross (CC)	http://csbio.unc.edu/CCstatus
Disease Ontology (DO)	http://disease-ontology.org
Diversity Outcross (DO)	http://cgd.jax.org/datasets/phenotype/SvensonDO.shtml
Drosophila Genome Database (FlyBase)	http://flybase.org
Ensembl	http://www.ensembl.org
*European Mouse Mutant Archive (EMMA)	http://www.emmanet.org
*Europhenome	http://www.europhenome.org
Foundational Model of Anatomy (FMA)	http://sig.biostr.washington.edu/projects/fm/AboutFM.html
Gene Ontology (GO)	http://www.geneontology.org
*Gene Weaver	http://www.GeneWeaver.org
Getting an Understanding of LOgical definitions (GULO)	http://compbio.charite.de/svn/hpo/trunk/src/tools/gulo
Human Phenotype Ontology (HP)	http://www.human-phenotype-ontology.org
International Classification of Diseases (ICD)	http://www.cdc.gov/nchs/icd.htm
International Knockout Mouse Consortium (IKMC)	http://www.knockoutmouse.org
*International Mouse Phenotyping Consortium (IMPC)	http://www.mousephenotype.org
International Pediatric and Congenital Cardiac Code (IPCCC)	http://www.ipccc.net
*Jackson Laboratory Mouse Repository (JAX Mice)	http://jaxmice.jax.org
KEGG Pathways Database	http://www.genome.jp/kegg/pathway.html
*Mammalian Phenotype Enrichment Analysis (MamPhEA)	http://evol.nhri.org.tw/phenome/index.jsp?platform=mmus
*Mammalian Phenotype Ontology (MP) browser	http://www.informatics.jax.org/searches/MP_form.shtml
*Mammalian Phenotype Ontology download from MGI site	ftp://ftp.informatics.jax.org/pub/reports/index.html#pheno
Mammalian Phenotype Requests Tracker	https://sourceforge.net/tracker/?atid=1109502&group_id=76834
MEDIC disease vocabulary	http://ctdbase.org/voc.go?type=disease
Medical Subject Headings (MeSH)	http://www.nlm.nih.gov/mesh/MBrowser.html
*MGI Mouse Genome Browser (Mouse GBrowse)	http://gbrowse.informatics.jax.org/cgi-bin/gbrowse/mouse_current
*MouseFinder	http://www.mousemodels.org
*MRC Harwell MouseBook	http://www.mousebook.org
*Mutant Mouse Regional Resource Centers (MMRRC)	http://www.mmrrc.org
National Center for Biotechnology Information (NCBI)	http://www.ncbi.nlm.nih.gov
*Online Mendelian Inheritance in Animals (OMIA)	http://omia.angis.org.au/home
Online Mendelian Inheritance in Man (OMIM)	http://www.omim.org
Ontology Lookup Service (OLS)	http://www.ebi.ac.uk/ontology-lookup/ontologyList.do
Open Biomedical Ontologies (OBO)	http://www.obofoundry.org
Orphanet	http://www.orpha.net/consor/cgi-bin
*PhenoHM	http://phenome.cchmc.org/phenoBrowser/Phenome
*PhenomeNet	http://phenomebrowser.net
*PhenomicDB	http://www.phenomicdb.de
Phenotype and Trait Ontology (PATO)	http://code.google.com/p/pato
*Rat Genome Database (RGD)	http://rgd.mcw.edu
Reactome	http://www.reactome.org
*Sanger Institute Mouse Resources Portal	http://www.sanger.ac.uk/mouseportal
Systematized Nomenclature of Medicine-Clinical Terms (SNOMED-CT)	http://www.ihtsdo.org/snomed-ct
*ToppGene: Candidate gene prioritization	http://toppgene.cchmc.org/prioritization.jsp
Uberon, cross-species anatomy ontology	http://obofoundry.org/wiki/index.php/UBERON:Main_Page
*UCSC genome browser	http://genome.ucsc.edu
UniProt-GOA (Gene Ontology Annotation)	http://www.ebi.ac.uk/GOA
*VeryGene	http://www.verygene.com
Zebrafish Database (ZFIN) phenotype example	http://zfin.org/action/phenotype/phenotype-statement?id=53698

MGI contains information on published spontaneous, induced, and genetically engineered mouse mutations (Table 1), as well as contributed or downloaded data from large-scale mouse mutagenesis projects, including ENU, gene trap, and knockout mutagenesis projects (see Table 3 for a list of mutagenesis projects with data integrated into MGI). All of these data are integrated with all of the other genomic, expression, function, tumor and pathway data in MGI to facilitate knowledge discovery and hypothesis building. The MGI website presents a mutant allele in all of its studied contexts, which is key for discerning multigenic disease models, genetic background effects, and allelic interactions. Tools also are available at MGI for users who wish to separate genotypes carrying single-gene mutations from more complex genotypes such as conditional genotypes or those carrying transgenes, mutations in multiple genes, or large genomic rearrangements.

**Table 3:** Major mutagenesis projects contributing data to MGI

Center	Reference	URL
**ENU Mutagenesis Projects**		
Australian Phenomics Facility at ANU	Nelms and Goodnow (2001)	http://www.apf.edu.au/about/projects/index.shtml
Cardiovascular Development Consortium (CvDC)	Kaltman et al. (2010)	http://www.devbio.pitt.edu/research/mouse_muta.html
Heart, Lung, Blood & Sleep Center (HLBS)	Svenson et al. (2003)	http://pga.jax.org/protocol_008.html
Helmholtz Zentrum Munchen IDG (formerly GSF)	Hrabé de Angelis et al. (2000)	http://www.helmholtz-muenchen.de/en/idg/mouse-genetics/large-scale-mutagenesis/enu-mutagenesis/
Mouse Mutagenesis for Developmental Defects (BCM)	Kile et al. (2003)	http://www.mouse-genome.bcm.tmc.edu/ENU/MutagenesisProj.asp
MRC Harwell Mutagenesis Project	Nolan et al. (2000)	http://www.har.mrc.ac.uk/research/mutagenesis/
Mutagenix	Hoebe and Beutler (2005)	http://mutagenetix.utsouthwestern.edu/
Neuroscience Mutagenesis Consortium (NMICE)	Goldowitz et al. (2004)	http://www.neuromice.org/About/ParticipatingCenters.jsp
Reproductive Genomics (ReproGenomics)	Lessard et al. (2004)	http://reproductivegenomics.jax.org
RIKEN Japan	Gondo et al. (2010)	http://www.brc.riken.jp/lab/gsc/mouse/
Sloan-Kettering Mouse Project	Anderson (2000)	https://mouse.mskcc.org/mutant/mutantBase.php
Toronto Centre for Modeling Human Disease (CMHD)	Cordes (2005)	http://www.cmhd.ca/enu_mutagenesis
**Gene Trap Projects (reported in IGTC**, www.genetrap.org)		
BayGenomics	Stryke et al. (2003)	http://www.mmrrc.org/catalog/overview_BG.php
European Mouse Mutagenesis Consortium (EUCOMM)	Friedel et al. (2007)	http://www.helmholtz-muenchen.de/en/idg/mouse-genetics/large-scale-mutagenesis/eucomm
Exchangeable Gene Trap Clones (EGTC)	Araki et al. (1999)	http://egtc.jp
German Gene Trap Consortium	Wiles et al. (2000)	http://www.helmholtz-muenchen.de/en/idg/mouse-genetics/large-scale-mutagenesis/german-gene-trap-consortium
Lexicon Genetics OMNIBANK	Zambrowicz et al. (1998)	Resource now available through TIGM
RIKEN BioResource Center	Matsuda et al. (2004)	http://www2.brc.riken.jp/lab/mouse_es/index.html.en
Sanger Institute Gene Trap Resource	Guo et al. (2004)	http://www.sanger.ac.uk/resources/mouse/sigtr/
Soriano Lab Gene Trap Database	Friedel and Soriano (2010)	http://research.mssm.edu/soriano/lab/gene_trap.html
The Institute for Genomic Medicine (TIGM)	Hansen et al. (2008)	http://www.tigm.org/
TIGEM-IRBM Gene Trap	Roma et al. (2008)	http://genetrap.tigem.it/public/index.php
Toronto Centre for Modeling Human Disease (CMHD)	To et al. (2004)	http://www.cmhd.ca/genetrap
**Knockout Mouse Projects**		
Deltagen	Moore (2005)	http://www.deltagen.com
European Mouse Mutagenesis Consortium (EUCOMM)	Auwerx et al. (2004)	www.eucomm.org
European Mouse Mutagenesis Consortium Tools (EUCOMMTools)	Skarnes et al. (2011)	www.eucommtools.org
Knockout Mouse Project (KOMP)	Austin et al. (2004)	http://www.knockoutmouse.org
Lexicon Genetics	Friddle et al. (2003)	http://www.lexicon-genetics.com
North American Conditional Mouse Mutagenesis (NorCOMM)	Collins et al. (2007a)	http://www.norcomm.org
The Institute for Genomic Medicine (TIGM)	Collins et al. (2007b)	http://www.tigm.org

This is a not an exhaustive list of major projects

**Fig. 2:**
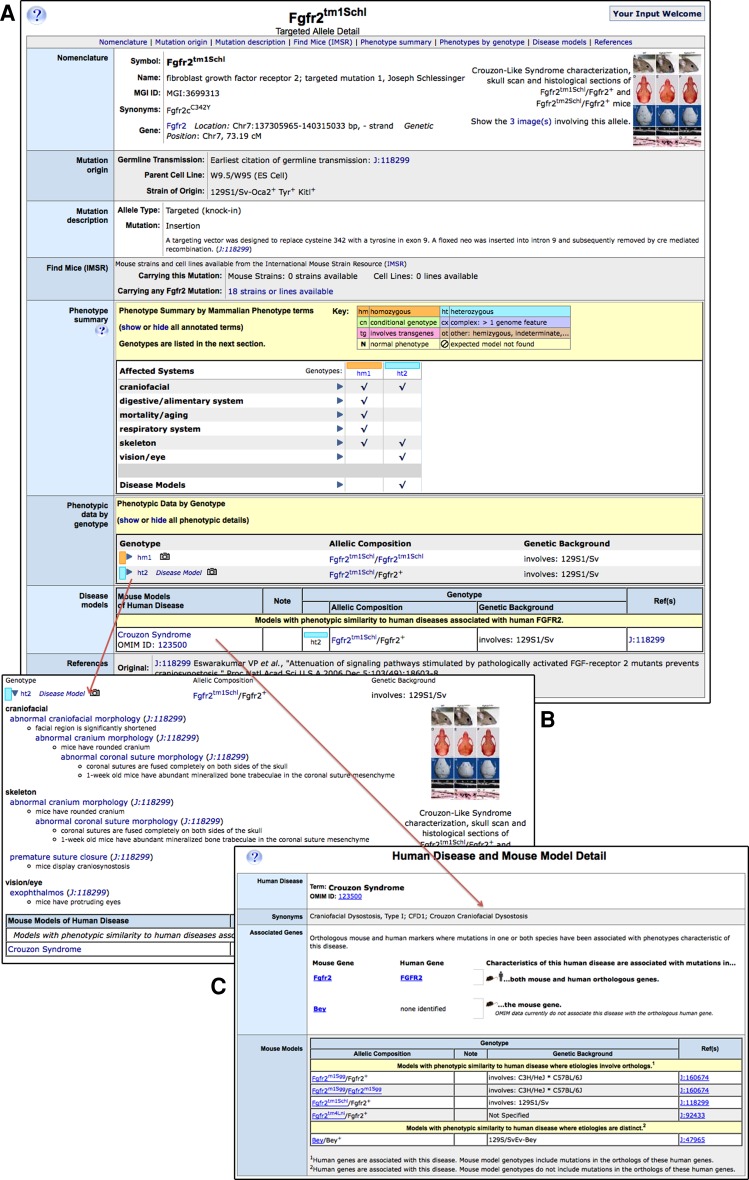
Example of a MGI allele detail page and associated Human Disease and Mouse Model detail page. **a** The *Fgfr2* ^*tm1Schl*^ (a mutation in the fibroblast growth factor receptor 2 gene, the first targeted mutation from the laboratory of Joseph Schlessinger) allele record shows nomenclature, a molecular description of the mutation, a phenotype summary organized by systems affected in tabular format to facilitate cross-genotype comparisons, a phenotype data by genotype section, a human disease model section, and associated references. **b** The phenotype by genotype section expands to reveal details, including further description and citations. Two different cohorts of mice carrying this allele have been analyzed, but only one has been asserted to be a model of the human disease Crouzon Syndrome (OMIM: 123500); links to both the MGI Human Disease and Mouse Model Detail page (**c**) and to OMIM are provided

Figure 2 shows an example of MP annotations to genotypes involving the *Fgfr2* ^*tm1Schl*^ mutant allele (a mutation in the fibroblast growth factor receptor 2 gene, the first targeted mutation of this gene from the laboratory of Joseph Schlessinger). Phenotype data are viewed in a matrix summary format to facilitate comparison of multiple genotypes and genetic backgrounds or by genotype. Clicking the links in these sections leads to an expanded view, including terms and additional details organized by physiological system, as well as mouse disease model data and images.

**Fig. 3:**
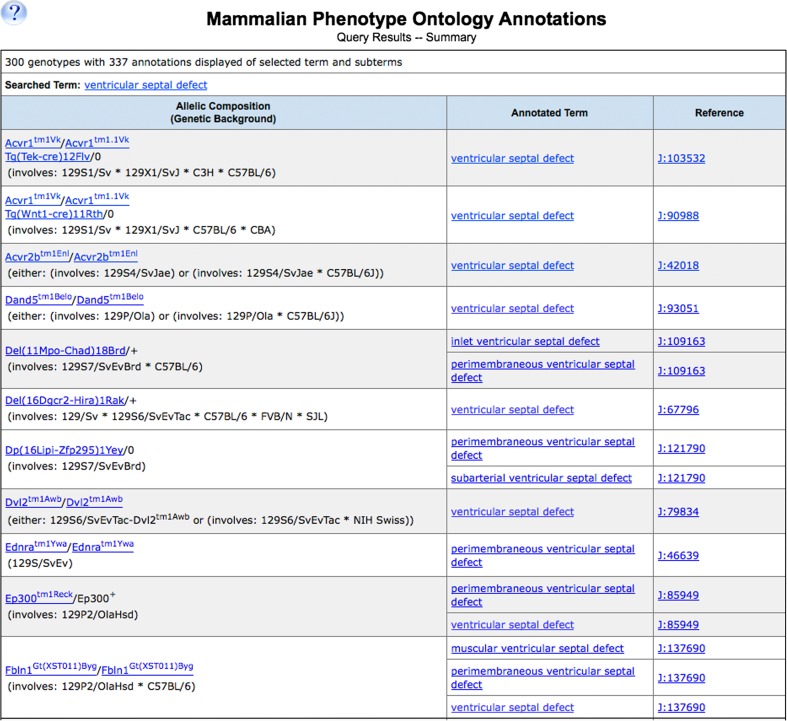
Example of a comprehensive MGI data search result using the term “ventricular septal defect.” Shown are samples of the results listed from this search. All 300 genotypes in MGI that are annotated to this term, or terms below this node in the MP, are returned. For example, genotypes annotated to the term “inlet ventricular septal defect,” a child term to “ventricular septal defect,” are seen in the results list

Phenotype data associated with specific terms are retrieved in a variety of ways. A MP term or ID entered in the Quick Search box on any MGI page will retrieve a list of genes, alleles, and vocabulary terms. MP terms entered in the Phenotype/Human Disease section of the advanced Genes and Markers Query Form or Phenotypes Query Form return genes or alleles associated with genotypes annotated to that term. Selecting terms in the MP Browser displays links at the term level to genotypes annotated to that term or any child of that term. For example, a search in MGI using either the identifier “MP:0010402” or the term name “ventricular septal defect” returns a list of 300 genotypes with 337 annotations representing 311 matching alleles in 232 genes, transgenes, and markers (Fig. 3). These results include annotations to terms listed below “ventricular septal defect” in the hierarchy such as “inlet ventricular septal defect” and “perimembraneous ventricular septal defect.” Thus, use of an ontology allows the retrieval of all information associated with a term and its children.

Phenotype data also are retrieved from the MGI Batch Query Form and the MGI BioMart. MGI also maintains a suite of public reports containing phenotype data for download. Using MGI’s public reports and web services, MGI data can be exported to a variety of other data providers such as NCBI, EBI, UCSC, OMIM, and mouse mutant repositories, where they are incorporated to enrich those resources.

## Relationship of the Mammalian Phenotype Ontology to other ontologies for model organism phenotypes and human disease data

Comparing phenotypes among organisms as well as against human phenotypes (and thereby with human disease) makes it possible to discover commonalities of gene function, pathways, and mechanisms. Because all organisms currently have significant gaps in the experimental knowledge of mutations and phenotypes for all genes and in the understanding of the function and interactions for each gene, comparative analyses can provide clues and direction for new experimental validation and research avenues.

At present, there is no universal phenotype ontology for all species that could easily facilitate comparative phenotyping. For mammals, the MP is widely accepted and applied (see above and Table 2). For human, the Human Phenotype Ontology (HP, http://www.human-phenotype-ontology.org) (Robinson et al. 2008), also a precomposed ontology, is actively being developed. For other model organisms, approaches vary, from species-specific vocabulary lists (e.g., in FlyBase, the *Drosophila* model organism database, http://flybase.org) to the “EQ” (entity + quality) approach (e.g., in ZFIN, the Zebrafish Information Network). In the EQ approach, terms are composed *de novo* at the time of annotation using an “entity” term found in other existing ontologies [e.g., Gene Ontology (GO, http://www.geneontology.org), Chemical Entities of Biological Interest (ChEBI, http://www.ebi.ac.uk/chebi)] plus a “quality” from the Phenotype and Trait Ontology (PATO, http://code.google.com/p/pato) (Gkoutos et al. 2005) that provides the term modifier (e.g., pale, enlarged, absent). For example, ZFIN combines the anatomy term “pericardium” (ZFA:0000054) with the PATO term “edematous” (PATO:0001450) to create a complete phenotype (EQ) statement “pericardium edematous, abnormal ” (http://zfin.org/action/phenotype/phenotype-statement?id=53698). The MP Ontology contains a single precomposed term “pericardial edema” (MP:0001787).

Mappings between terms of phenotype ontologies harmonize these different approaches. For the MP, direct mappings to other precomposed phenotype ontologies such as the HP, or indirect mappings of MP terms to EQ statements (Mungall et al. 2010) are used. The EQ mapping consists of developing a “logical definition” in ontological parlance. Logical definitions for MP and HP can be combined with annotations from other species databases that use EQ statements to describe phenotypes, making multispecies phenotype data integration and comparisons possible (Mungall et al. 2010; Washington et al. 2009). Importantly, logical definitions enhance the MP by establishing relationships of terms to a wider suite of interoperating ontologies. However, aggregate terms such as hydrocephaly or glomerular crescent require representation as multiple EQ statements, diminishing the meaning and recognition of these scientific/clinical terminologies.

Ontologies developed as annotation tools (e.g., MP, HP, and GO) are improved by mapping concepts to a common reference framework based on existing standard ontologies (such as the global anatomy ontology Uberon, http://obofoundry.org/wiki/index.php/UBERON:Main_Page) (Mungall et al. 2012). Maintenance of ontologies with multiple inheritance pathways becomes increasingly difficult with increased size and complexity, and they are particularly difficult to view for missing terms when additions are largely dictated by the need of curation or projects to define new terms. Missing terms can be logically assumed (i.e., if there is a term “increased X,” the converse term “decreased X” should exist), although such terms might not be biologically relevant phenotypes. Automatic reasoners, software tools that infer the positions of terms in a subsumption hierarchy based on logical definitions, have been exploited to identify missing or erroneous relationships and detect omissions in ontologies (Mungall et al. 2011). To this end, the tool GULO (Getting an Understanding of LOgical definitions, http://compbio.charite.de/svn/hpo/trunk/src/tools/gulo) (Köhler et al. 2011) was applied to refine the MP. Based on the results of reasoner analysis, MP added over 300 new child–parent relationships. This work also uncovered discrepancies in reference ontologies used to construct logical definitions and errors in assignment of EQ statements. Therefore, the use of logical definitions, coupled with software tool reasoners, automates some aspects of ontology review for completeness and term placement in the DAG, although manual verification is needed to ensure accuracy and to place aggregate terms.

A combination of EQ statements and precomposed MP terms can reciprocally meet the needs of automated phenotype annotation pipelines and biomedical researchers interested in exploring data. For example, Europhenome describes parameters tested through the phenotyping pipeline using EQ statements. An eye dysmorphology parameter defined by “eye” (MA:0000261) and “size” (PATO:0000117) defines the test itself, and a phenotypic observation may then be “eye” and “decreased size” (PATO:0000587). This EQ combination corresponds to the MP term “microphthalmia” (MP:0001297), or small eye, a term familiar to scientists and clinicians and used by Europhenome in its web interface and BioMart (Beck et al. 2009; Morgan et al. 2010).

## Integration with ongoing and new systematic phenotyping efforts

Systematic phenotyping, where many centers apply common phenotyping protocols under the same conditions (e.g., age, sex), can provide high-quality data sets for analysis and comparison. This interinstitution standardization of phenotype testing was experimented with and analyzed extensively in the last decade in the European projects of EUMORPHIA (Brown et al. 2005; Mandillo et al. 2008) and EUMODIC (Beck et al. 2009; Morgan et al. 2010). The role of EUMODIC was to generate phenotype data from the first 500 mutant mouse knockout lines derived from the International Knockout Mouse Project (IKMC, http://www.knockoutmouse.org) (Ringwald et al. 2011; Skarnes et al. 2011) using standardized protocols and pipelines. The results produced by these centers are reported through the Europhenome database (Morgan et al. 2010) and are available through a web interface or through the IKMC BioMart (Oakley et al. 2011). In addition, data generated by the Sanger Institute are available from their mouse resources portal. Both of these resources perform statistical comparisons of experimentally generated mutant mouse phenotype data with control phenotype data to identify significant phenodeviants. Significant results are stored and the relevant MP term for the test result is automatically applied to the mutant line. In addition to automated pipelines, data also are analyzed manually at the Sanger Institute and phenodeviant calls and MP terms are assigned to these results. Both the Europhenome and Sanger Institute’s Mouse Resource Portal sites allow searching and browsing for phenodeviant data using MP terms [see review of both of these efforts in Ayadi et al. (2012)].

MGI is undertaking the importation of these data to integrate them with all the resources that MGI offers. Because these phenotype data are already associated with MP terms, as well as official gene, allele, and strain nomenclature and other standardized parameters, importation is automatable and thus reduces the need for further curation. Similarly, MGI can effectively work with data sets of increasing size, such as those expected from the IMPC, which has a stated goal to carry out high-throughput phenotyping for over 20,000 mutant mouse lines to determine the function of every gene in the mouse genome (Brown and Moore 2012).

## Use of phenotype ontologies and mouse phenotype and disease annotations in research and online tools

Mouse genotypes in MGI are annotated to human disease terms from OMIM (Amberger et al. 2011) when an author demonstrates that the phenotype mimics the human disease state. For example, the *Fgfr2* ^*tm1Schl*^ allele is a model of the human disease Crouzon Syndrome (OMIM ID:123500) (Fig. 2) (Eswarakumar et al. 2006). Links from MGI mutant allele details to both the OMIM record describing this condition in humans and to MGI’s Human Disease and Mouse Model web pages are provided. These models are searched using the OMIM term or ID from the Quick Search Box on any MGI page or the advanced Genes and Markers Query Form or Phenotypes Query Form, or they may be browsed on the Human Disease Vocabulary browser. OMIM is used as MGI’s source of human genetic disease terms because it provides associated detailed descriptions of human disease and clinical synopses, associates OMIM disease records to human genes, and is recognized and frequently used by clinicians and biomedical researchers as an authoritative information source.

Given the exponentially increasing amount of complex mouse phenotype and human disease model data in MGI and elsewhere and that these data are stored in model organism databases using different methods, computational tools are required that will lead to better data mining and comparison of phenotypic data across different species. There are a number of approaches using lexical matching or ontology mapping of phenotype or clinical terms that map phenotypic similarity between mouse and human genes and variants and suggest candidate genes for human diseases [e.g., PhenomicDB, http://www.phenomicdb.de (Groth et al. 2010); PhenoHM, http://phenome.cchmc.org/phenoBrowser/Phenome (Sardana et al. 2010); MouseFinder, http://www.mousemodels.org (Chen et al. 2012); PhenomeNet, http://phenomebrowser.net (Hoehndorf et al. 2011; Gkoutos et al. 2012; and reviewed in Schofield et al. 2012)].

In addition to comparing data across species, MP annotated phenotype data are used as a parameter by a number of web tools that integrate published and high-throughput data to facilitate gene discovery via enrichment analysis of gene sets or to identify candidate genes for QTL. Among the tools for enrichment analysis are MamPhea (http://evol.nhri.org.tw/phenome/index.jsp?platform=mmus) (Weng and Liao 2010), which enables gene enrichment analysis of genes from multiple species based exclusively on MP annotations from mouse, and ToppGene (http://toppgene.cchmc.org/prioritization.jsp) (Chen et al. 2009), a gene enrichment tool that uses MP as one of many parameters for sorting gene sets.

Other resources include Gene Weaver (http://www.GeneWeaver.org) (Baker et al. 2012), which integrates sets of biological functions (GO), their relations to mutant phenotypes through the MP, KEGG pathways (http://www.genome.jp/kegg/pathway.html), QTL data, and more. VeryGene (http://www.verygene.com) (Yang et al. 2011) links tissue-specific gene expression data to data on gene function (GO), Reactome (http://www.reactome.org), KEGG pathways, MP annotations, disease associations, and targeting drugs. Among the web tools for candidate gene identification is the AnnotQTL tool (http://annotqtl.genouest.org) (Lecerf et al. 2011), which adds mouse MP annotations, as well as mouse and human gene function (GO) annotations, to genes in an identified QTL interval region to assist in predicting candidate genes.

Using the tools described above and elsewhere, a number of recent studies highlight the use of mouse MP annotations in the identification or validation of candidate gene sets in human disease and mouse studies:

- Dickerson et al. (2011) identified 1,965 human disease genes from OMIM’s morbid map and separated them according to whether the knockout phenotype of the mouse ortholog was lethal (essential) or viable from phenotype data coded to the MP in MGI. Human genes in this set with mouse orthologs having a lethal phenotype are over-represented among disease genes associated with cancer and highly connected in protein–protein interaction networks.
- Russell et al. (2012) discovered novel candidate genes for congenital diaphragmatic hernia by expression profiling of mouse embryonic diaphragm, then applying gene enrichment analysis on this identified set with MGI annotated data of muscle development and metabolism terms in the GO and abnormal muscle and cardiovascular phenotype terms from the MP ontology. Twenty-seven new candidate genes were identified. One candidate gene, pre-B-cell leukemia transcription factor one (*Pbx1*), when mutated, results in a range of previously undetected diaphragmatic defects in mice.
- Meehan et al. (2011) used MGI phenotype data associated with MP terms and mouse model data to create a set of similarly annotated genes/genotypes likely to have previously uncharacterized autistic-like phenotypes. The implicated genes considerably overlapped with a set of over 300 human genes associated with human autism spectrum disorder due to small, rare copy number variants (CNVs, Pinto et al. 2010). Similarly, Gai et al. (2012) identified 12 MP ontology term annotations that are significantly enriched in genes overlapping inherited rare autism CNVs and are consistent with observable phenotypes associated with human autism spectrum disorder behaviors.
- Shaikh et al. (2011) identified a group of genes enriched in human developmental delay-associated CNVs, which when disrupted in mice, result in specific nervous system phenotypes. The most significant term annotated to these genes was an abnormal nervous system white matter tract phenotype, which was used to narrow the candidate gene set for further analysis.
- Bayés et al. (2011) identified 1,461 proteins present in human neocortex postsynaptic density. Mutations in 199 of these genes were associated with human neurological diseases in OMIM. Enrichment analysis revealed 77 MP terms, including cognitive and motor phenotypes associated with mutations in the mouse orthologs that revealed new candidate genes. A similar gene enrichment result was shown using the Human Phenotype Ontology annotations derived from OMIM.
- Hageman et al. (2011) used MP annotations to kidney phenotypes to narrow the genomic intervals and find candidate genes for QTL affecting the urinary albumin-to-creatinine ratio in mice.

Thus, the predictive value of mouse mutant phenotypes in identifying new candidate genes assists researchers in revealing the complex nature of human diseases.

## Summary and future prospects

The MP ontology continues to evolve and expand to robustly describe phenotypes. New terms and structural refinements are incorporated as required by phenotype annotation efforts at MGI and other databases, phenotyping centers, mutagenesis projects, investigator research, and review by biological domain experts. MGI curates information on published mouse mutations and electronically imports phenotype and disease model information from other sources.

MGI continues to adapt as new data drive database infrastructure and as public data presentation changes. For example, changes are already underway to accommodate Europhenome and the Sanger Institute’s large-scale phenotype data derived from targeted knockout mutations, as well as future IMPC phenotype data. Additional new sources of mouse allele, variant, and phenotype data will arise from the Collaborative Cross (CC, http://csbio.unc.edu/CCstatus) (Churchill et al 2004; Threadgill and Churchill 2012) and the Diversity Outcross (DO, http://cgd.jax.org/datasets/phenotype/SvensonDO.shtml) (Svenson et al. 2012), as well as mutations induced by engineered zinc finger nucleases (Osiak et al. 2011). Other mutation-generation techniques, including transposon-induced mutations (cf. Largaespada 2009; Liang et al. 2009; Takeda et al. 2007; Wang et al. 2008), and the detection by NexGen and whole-exome sequencing of significant numbers of previously undetectable ENU mutations (cf. Arnold et al. 2011; Boles et al. 2009; Guryev and Cuppen 2009; Sun et al. 2012) will further increase the genomic mutations and phenotypic data that require MP and nomenclature standards for integration with existing data. The promise of integrating these many streams of phenotype data with a robust MP ontology will enable a growing reservoir of standardized data for data mining, gene set enrichment studies, candidate disease model identification, and validation of computational predictions.

Many challenges remain in the ability to use computational tools to analyze and compare data from human clinical and mouse phenotype resources. Human GO data are freely available via the Universal Protein Resource GO Annotations (UniProt-GOA, MP Ontology http://www.ebi.ac.uk/GOA) (Dimmer et al. 2012), but genetic, disease, and clinical data are scattered in many databases with differing formats and accessibility, and many resources are not maintained in a computational-friendly format (Küntzer et al. 2010). The HP, now being adopted by resources such as NCBI, is available for standardization of human clinical symptoms (Robinson et al. 2008) and is mapped to OMIM disease records. Logical definitions derived for the HP are mapped to similar ontologies such as the MP (Mungall et al. 2011).

The HP is only one part of the infrastructure needed for human disease data management, however. A comprehensive disease ontology with descriptions and definitions of disease terms in the context of observable clinical features, including a mapping to other phenotype ontologies such as HP and/or MP, is required to maximize the interoperability and computational access to the wide range of human disease data. Current vocabularies for human disease have a number of drawbacks that prevent their wide adoption as a robust source for human disease annotation. OMIM, while an excellent source of text descriptions of disease, lacks a hierarchical structure and is limited to Mendelian disease. The international classification of disease (ICD, http://www.nlm.nih.gov/mesh/MBrowser.html) is designed for physician billing codes and thus is confounded by many nondisease terms such as those for injury and infection. The Systematized Nomenclature of Medicine—Clinical Terms (SNOMED-CT, http://www.ihtsdo.org/snomed-ct) must be licensed for use by country or affiliation and is thus not a publicly available resource.

Several nascent efforts that are developing human disease ontologies/vocabularies are underway. These include the Disease Ontology (DO, http://disease-ontology.org) (Schriml et al. 2012), MEDIC (http://ctdbase.org/voc.go?type=disease) (Davis et al. 2012), and Orphanet (http://www.orpha.net/consor/cgi-bin) (Rath et al. 2012). In addition, the Medical Subject Headings (MeSH)-disease branch at the U.S. National Library of Medicine (Nelson et al. 2004) is increasingly incorporating OMIM disease terms. Ultimately, the successful growth and maturation of one or more of these or other proposed disease ontologies and vocabularies should lead to greater interoperability of human genetic, disease, and clinical data among the scattered resources, as well as integration with model organism data. Adoption of semantic and syntactic standards by the human clinical community will facilitate integration of data from a multitude of resources and allow the ability to compute over many data sets, as has been demonstrated for mouse genetic and phenotype data via the MP.

## Footnotes

1 This number refers only to mutant alleles that are “in mice.” The number of alleles known exceeds 730,000 when also counting those alleles that exist only as targeted or gene-trapped mutations in ES cell lines.
